# A Cascade BP Neural Network Tuned PID Controller for a High-Voltage Cable-Stripping Robot

**DOI:** 10.3390/mi14030689

**Published:** 2023-03-20

**Authors:** Jun Zhong, Shaoguang Hu, Zhichao Wang, Zhenfeng Han

**Affiliations:** 1 College of Mechanical & Electrical Engineering, Hohai University, Changzhou 213022, China; 2 HRG Institute (HeFei) of International Innovation, Hefei 230000, China

**Keywords:** cable-stripping robot, power grid, BP Neural Network, PID, cascade controller

## Abstract

A 10 kV distribution network is a crucial piece of infrastructure to guarantee enterprises’ and households’ access to electricity. Stripping cables is one of many power grid maintenance procedures that are now quickly expanding. However, typical cable-stripping procedures are manual and harmful to workers. Although numerous automated solutions for grid maintenance have been created, none of them focus on cable stripping, and most of them have large dimensions to guarantee multi-functions. In this paper, a new cable-stripping robot for the 10 kV power system is introduced. The design of a live working cable-stripping robot that is appropriate for installing insulating rods is introduced, taking into account the working environment of 10 kV overhead lines and the structural characteristics of overhead cables. The robot is managed by an auxiliary remote control device. A cascade PID control technology based on the back propagation neural network (BPNN) method was developed, as the stripper robot’s whole system is nonlinear and the traditional PID controller lacked robustness and adaptability in complex circumstances. To validate the structural feasibility of the cable-stripping robot, as well as the working stability and adaptability of the BPNN–PID controller, a 95 mm^2^ cable-stripping experiment are carried out. A comparison of the BPNN–PID controller with the traditional PID method revealed that the BPNN–PID controller has a greater capacity for speed tracking and system stability. This robot demonstrated its ability to replace manual stripping procedures and will be used for practical routine power maintenance.

## 1. Introduction

Power supply systems are playing a bigger role in residential energy consumption, factory power supply, and other areas as society as a whole develops. People pay more attention to the stability and dependability of the power supply system, especially factory users [1,2]. Power outages significantly affect industrial production [3,4]. The stripping of cable insulation is a crucial activity in live line maintenance, particularly the job of crimping overhead line conductor tension tubes and connecting drainage lines [5,6,7]. The traditional manual cable-stripping approach, which is risky, laborious, physically taxing, and challenging to learn, is difficult for workers to utilize because of the great strength and hardness of polyethylene material [8]. The inner metal of overhead cables is easily damaged during the typical wire-stripping process, which uses mechanical knives and is bad for line transmission [9]. Furthermore, efficient operations cannot be achieved using the manual method, as the cutter head must be modified in accordance with cable diameters [10]. In this paper, a cable-stripping robot for live work is proposed. This robot can increase operational efficiency and decrease damage to overhead cables’ inner metal, both of which are crucial for the stability and dependability of power transmission in distribution networks.

In recent years, some scientists have studied stripping tools. Yan created a wire-stripping tool [11] that consists of a lower transmission component and an upper peeling portion for the manipulator’s end. The bottom transmission component is connected to the motor output shaft to cause the peeling device to rotate, which enables the wire-stripping operation. The higher peeling part can revolve around a wire’s central axis to cut the insulation layer of the wire. The lack of an insulation thickness sensing system, however, prevents correct feeding, which could harm the inner metal or result in a clean cut in the insulation layer. A wire-stripping robot to perform direct cutting was created [12]. The cage support, connecting rod assembly, actuator, and lead screw make up the majority of this wire-stripping robot’s construction. The cage support has twelve cutters installed, and a motor powers the screw nut mechanism, which moves the connecting rod assembly and creates tension in the cage support. The cutter then cuts into the wire and strips it along the wire’s radial direction. A dual arm cooperative robot was suggested by Zhang [13]. An actuator, insulating mechanism, sensor system, power supply system, and control system make up the robot’s system. Remote operation can be used to complete the high-voltage line stripping procedure. 

Despite the fact that numerous instruments and robots have been created to maintain electricity grids, they are often huge and not specifically designed for cable peeling. Therefore, a unique small wire-stripping robot needs to be developed. As the operating stripper robot is a complicated nonlinear system, stable system operation is essential for the stripper robot to successfully finish the strip. However, it is difficult to carry out adaptive control using the traditional PID control algorithm; therefore, a brand-new stripping robot is introduced in this paper, and its adaptive control scheme design is discussed as follows. The cable-stripping robot system is introduced in Section 2, the design of the cascade controller based on a BP neural network is discussed in Section 3, and experiments carried out to validate the robot and its BPNN tuned PID strategy are described in Section 4.

## 2. Description of the Cable-Stripping Robot

**Figure 1:**
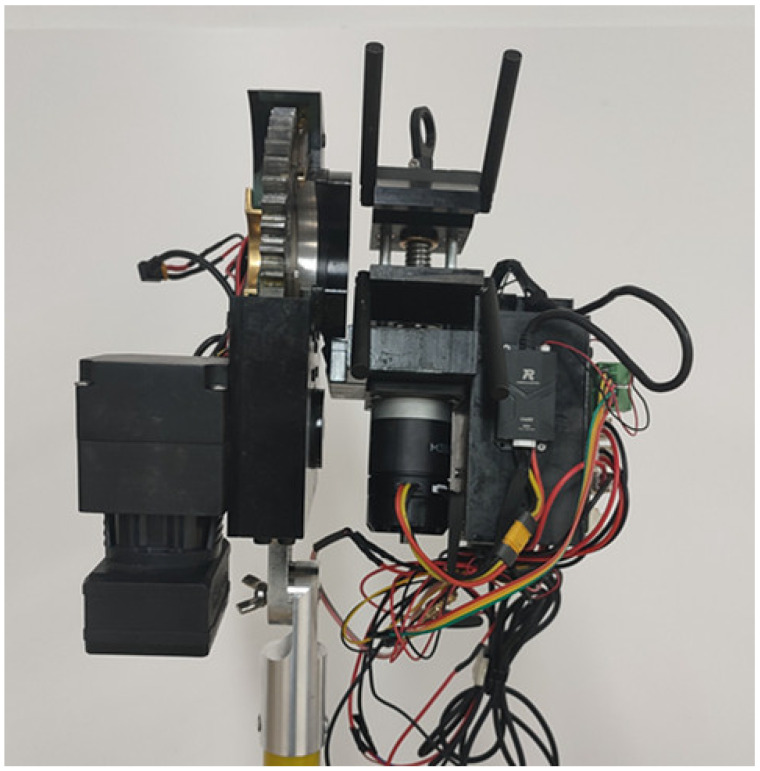
Prototype of the cable-stripping robot.

**Figure 2:**
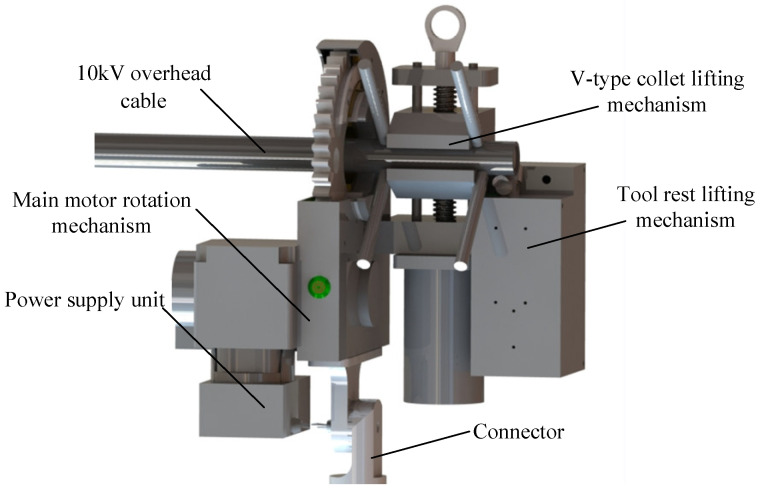
Structure of the cable-stripping robot.

Figure 1 and Figure 2 depict the cable-striping robot’s prototype and general structure, which were used in the actual operation of a 10 kV line. The robot consists of the main motor-rotating mechanism, the V-type collet lifting section, and the tool rest lifting component. The seat of the insulating rod’s quincunx holds the primary motor’s rotating mechanism in place. The tool rest lifting component is fixed to the collet mounting seat of the main motor rotating mechanism, and rotates around the overhead cable in the guide collet control box of the V-type collet lifting mechanism. The tool rest lifting mechanism has an infrared sensor built in, which enables automatic feed based on sensor detection results, ensuring the robot’s operational stability. The robot’s interior has specialized wire channels. The control line is a shielded twisted pair, which has good anti-electromagnetic interference properties.

The key parameters of the cable-stripping robot in stable operation are shown in Table 1.

**Figure 3:**
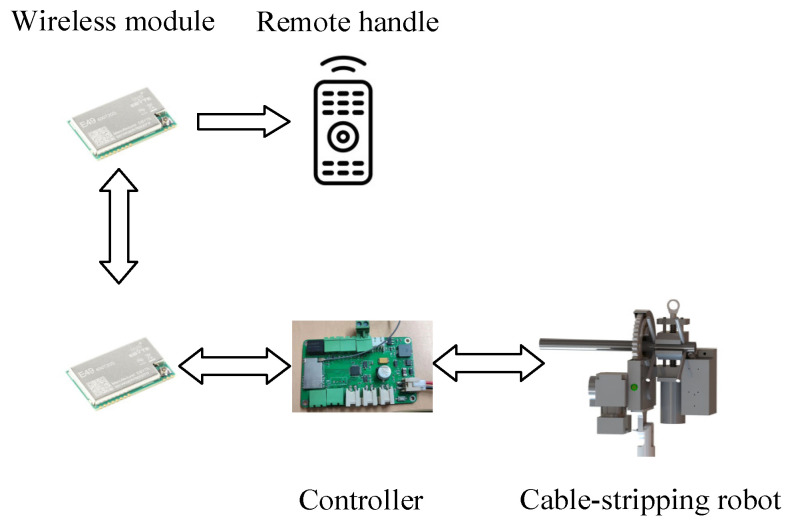
The entire cable-stripping robotic system.

Figure 3 depicts the full robotic system, which consists of a cable-stripping robot, a wireless remote handle, and an insulating rod. The system’s operational steps are as follows. First install the wire-stripping robot adapter at the end of the insulating rod. Next, make your way to the area near the overhead line. Radio frequency modules are used to connect the robot and the supplemental remote handle. The robot is given commands via the auxiliary remote-control device, which also directs it to carry out the necessary actions. The robot’s real-time data are sent to the supplementary remote handle through the wireless modules.

## 3. A Cascade BPNN–PID Controller for the Cable-Stripping Robot

### 3.1. General Scheme of the Robot Controller

**Figure 4:**
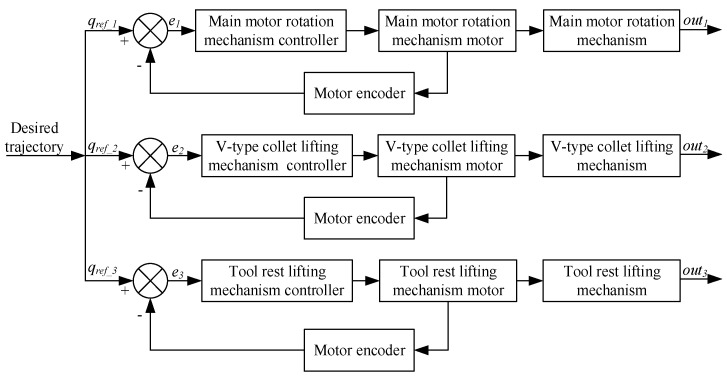
Motion control scheme of the cable- stripping robot.

In this design, the controller manages each motor speed separately to achieve precise control of each robot mechanism and accomplish the wire-stripping task. The PID approach, regulated by the BP neural network, is used to implement motor control. Figure 4 depicts the cable-stripping robot’s overall control architecture.

### 3.2. BP Neural Network

**Figure 5:**
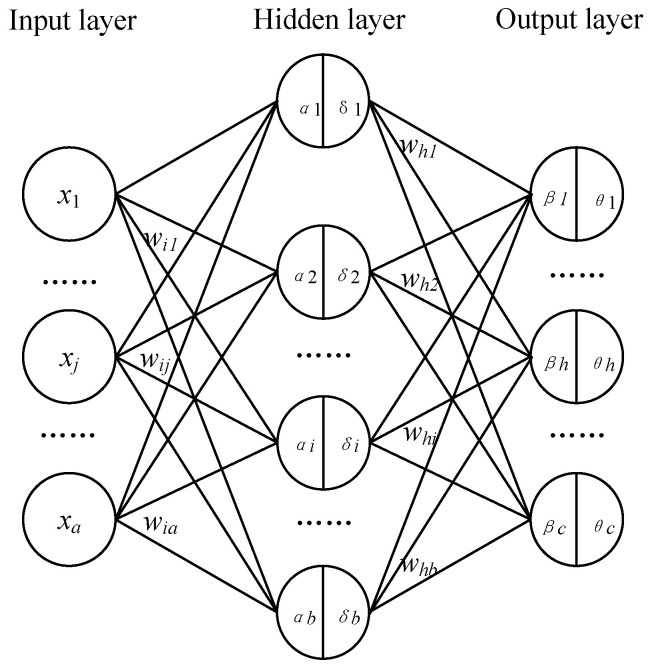
BP neural network with three layers.

A three-layer BP neural network comprising *a* input neurons *b* hidden neurons and *c* output neurons is depicted in Figure 5 [14]. The threshold of the *i*th neuron in the hidden layer is *δ_i_* and the weight from the input layer to the hidden layer is *w_ij_*. The threshold of the *h*th neuron in the output layer is set to *θ_h_*, and the weight from the hidden layer to the output layer is set to *w_hi_*.

The input of the *i*th neuron in the hidden layer can be obtained via the weighted sum of signals in the input layer.

(1) $$\alpha_{i}=\sum_{j=1}^{a}w_{ij}\cdot x_{j}$$

The input matrix in the hidden layer is expressed as

(2) $$\left[\begin{array}{c} \alpha_{1}\\ \alpha_{2}\\ \ldots \\ \alpha_{b} \end{array}\right]=\left[\begin{array}{cccc} w_{11} & w_{12} & \ldots & w_{1a}\\ w_{21} & w_{22} & \ldots & w_{2a}\\ \ldots & \ldots & \ldots & \ldots \\ w_{b1} & w_{b2} & \ldots & w_{ba} \end{array}\right]\left[\begin{array}{c} x_{1}\\ x_{2}\\ \ldots \\ x_{a} \end{array}\right]$$

The input of the output layer can be obtained as follows.

(3) $$\beta_{h}=\sum_{i=1}^{b}w_{hi}\cdot m_{i}$$

where *m_i_* is activation functions in the hidden layer.

Activation functions in the output layer are as follows.

(4) $$y_{h}=f(\beta_{h}-\theta_{h})$$

Define output error function as

(5) $$E_{k}=\frac{1}{2}{\sum_{h=1}^{c}\left(y_{h}-y_{h}^{*}\right)}^{2}$$

where *y_h_* is the actual output value, and y_h_* is the expected output value.

In summary, *w_hi_* first affects *β_h_*, then affects *y_h_*, and finally affects *Ek* [15]. Therefore, the weight correction Δ*w_hi_*(*n*) is

(6) $$\Delta w_{hi}=-\eta \frac{\partial E_{k}}{\partial w_{hi}}=-\eta \frac{\partial E_{k}}{\partial y_{h}}\cdot \frac{\partial y_{h}}{\partial \beta_{h}}\cdot \frac{\partial \beta_{h}}{\partial w_{hi}}=-\eta (y_{h}-y_{h}^{*})f^{\prime }(\beta_{h}-\theta_{h})m_{i}$$

The derivative of the activation function can be obtained

(7) $$f^{\prime }(\beta_{h}-\theta_{h})=f(\beta_{h}-\theta_{h})\cdot (1-f(\beta_{h}-\theta_{h}))=y_{h}\cdot (1-y_{h})$$

The weight increment from the hidden layer to the output layer is

(8) $$\Delta w_{hi}(n)=-\eta \frac{\partial E_{k}}{\partial w_{hi}}=-\eta (y_{h}-y_{h}^{*})y_{h}(1-y_{h})m_{i}$$

where *η* is the learning efficiency.

The weight *w_hi_*(*n* + 1) from the hidden layer to the output layer after adjustment is

(9) $$w_{hi}(n+1)=w_{hi}(n)+\Delta w_{hi}(n)$$

The correction value Δ*θ_h_*(*n*) of the threshold from the hidden layer to the output layer is

(10) $$\Delta \theta_{h}(n)=-\eta \frac{\partial E_{k}}{\partial \theta_{h}}=\eta (y_{h}-y_{h}^{*})y_{h}(1-y_{h})$$

After adjustment, the threshold value *θ_h_*(*n* + 1) from the hidden layer to the output layer is

(11) $$\theta_{h}(n+1)=\theta_{h}(n)+\Delta \theta_{h}(n)$$

Similarly, the weight correction Δ*w_ij_*(*n*) and threshold correction *δ_i_*(*n*) from the input layer to the hidden layer can be written as follows.

(12) $$\Delta w_{ij}(n)=-\eta \frac{\partial E_{k}}{\partial w_{ij}}=-\eta (y_{h}-y_{h}^{*})y_{h}(1-y_{h})w_{ij}m_{i}(1-m_{i})x_{i}$$

(13) $$\Delta \delta_{i}(n)=-\eta \frac{\partial E_{k}}{\partial \delta_{i}}=\eta (y_{h}-y_{h}^{*})y_{h}(1-y_{h})w_{ij}m_{i}(1-m_{i})$$

*w_ij_*(*n* + 1), the weight from the input layer to the hidden layer after adjustment, is

(14) $$w_{ij}(n+1)=w_{ij}(n)+\Delta w_{ij}(n)$$

The adjusted threshold *δ_i_*(*n* + 1) from the input layer to the hidden layer is

(15) $$\theta_{i}(n+1)=\theta_{i}(n)+\Delta \theta_{i}(n)$$

Weight and threshold corrections are iterated until the end of the iteration.

### 3.3. Cascade PID Controller Tuned via BP Neural Network

**Figure 6:**
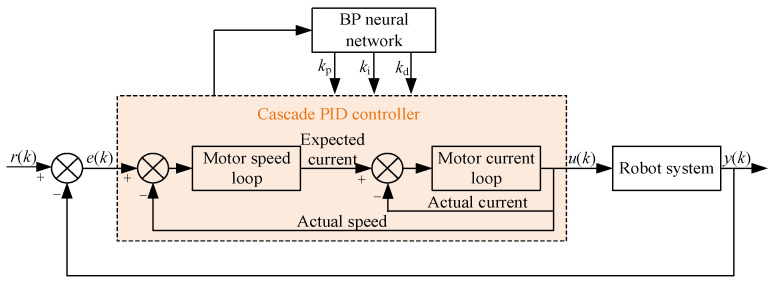
Cascade BPNN–PID controller.

According to tracking errors, the BP neural network modifies the PID parameters [16,17,18]. Figure 6 depicts the BP neural network’s cascade PID control principle.

The weight *w_hi_* from the input layer to the hidden layer, the weight *w_ij_* from the hidden layer to the output layer, and the learning efficiency *η* must all be determined in order to adjust the cascade PID controller using BPNN. The neural network layout in Figure 6 is condensed to nine input neurons and four output neurons in order to enhance the algorithm’s convergence. The layers that make up the input are *e*(*k*), *e*(*k* − 1), *e*(*k* − 2), *y*(*k*), *y*(*k* − 1), *r*(*k*), *u*(*k* − 1), the hidden layer and output layer weight coefficient (*k* − 2), and the hidden layer and output layer weight coefficient (*k* − 1); the layers that make up the output are *u*(*k*), *k*_p,_ *k*_i_ and *k*_d_ [19].

The following describes how the BPNN tuned cascade PID control strategy iterates.

(1) BPNN calculates inputs and outputs of each layer, and finally takes the output neuron of the output layer as the parameters *k*_p,_ *k*_i_, and *k*_d_ of the PID algorithm;
(2) The controller outputs *u*(*k*) through PID calculation, and finally the robot outputs *y*(*k*);
(3) Calculate the weight *w_hi_*(*k* + 1) from the input layer to the hidden layer and the weight *w_ij_*(*k* + 1) from the hidden layer to the output layer;
(4) Calculate output quantities *k*_p,_ *k*_i_, and *k*_d_ in the output layer;
(5) Calculate the output control quantity *u*(*k*) of BPNN through the PID cascade controller;
(6) Set *k* = *k* + 1 and return to step (2).

## 4. Experiments and Analysis

**Figure 7:**
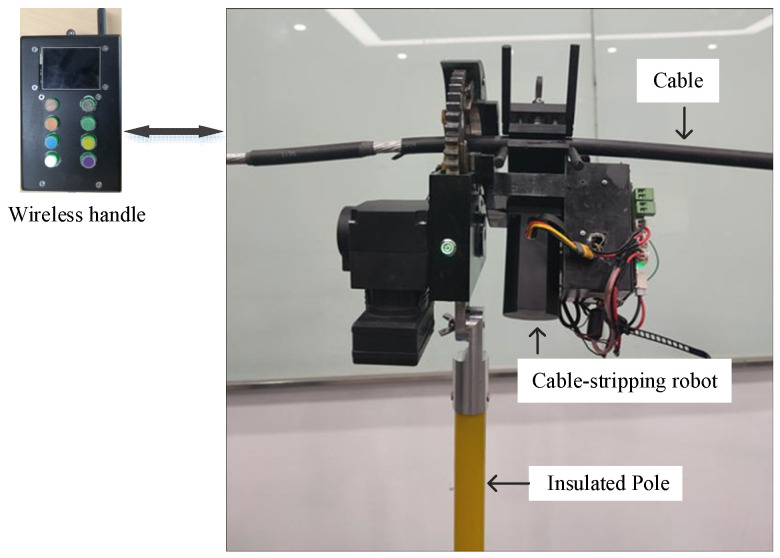
Experimental setup of the cable-stripping robot.

**Figure 8:**
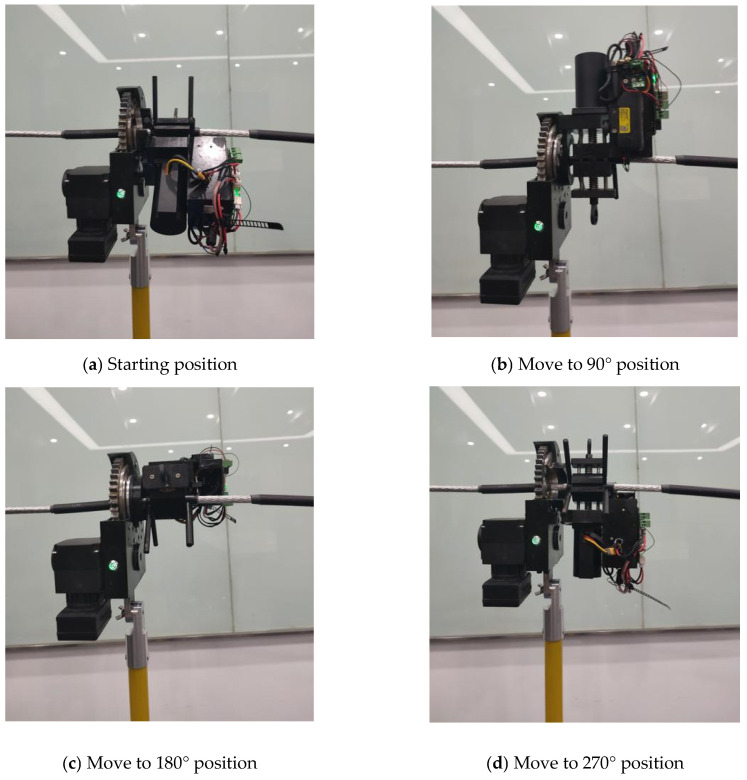
Stripping process of the robot.

After designing the mechanical system and control system of the cable-stripping robot, the experimental setup of the cable-stripping robot is built, as shown in Figure 7. The 95 mm^2^ cross-sectional area wire utilized in these experiments is frequently used in distribution networks. In Figure 8, the experimental procedure is displayed.

**Figure 9:**
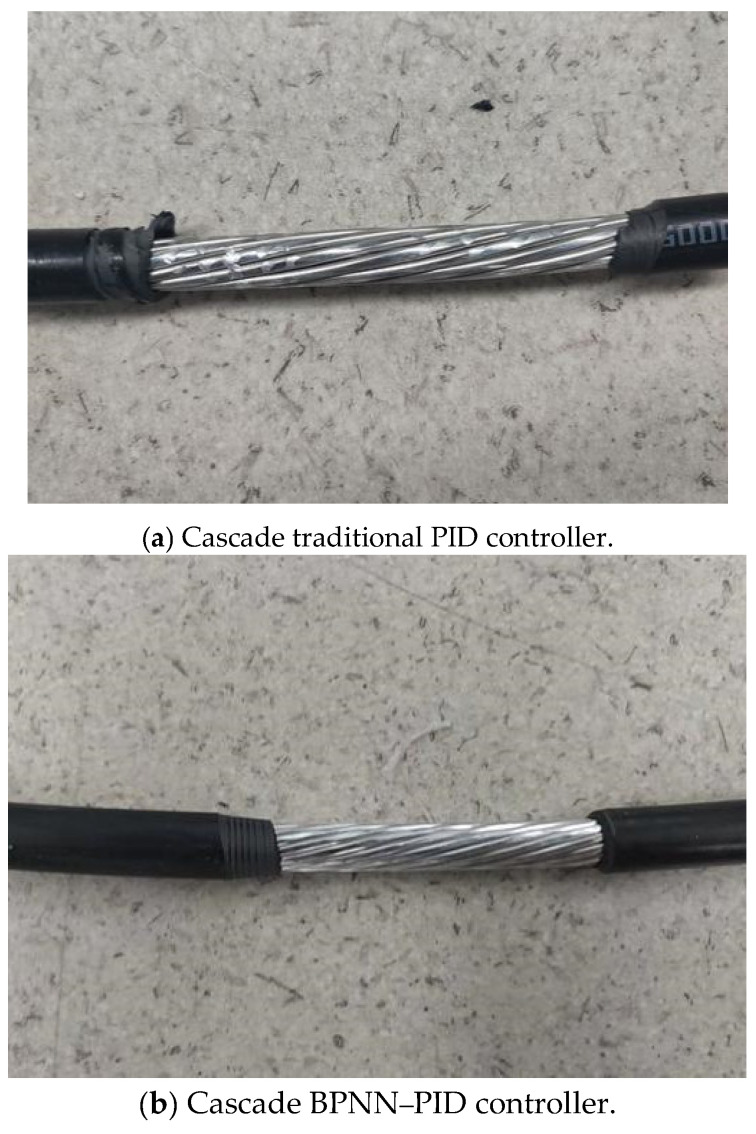
Stripped appearance using different controllers.

Figure 9 depicts how the stripped cable skin appeared using different controllers. Evidently, the robot’s cascade BPNN–PID controller provided superior stripping performance compared with the traditional cascade PID algorithm; Figure 9b shows less cable skin damage as a result.

**Figure 10:**
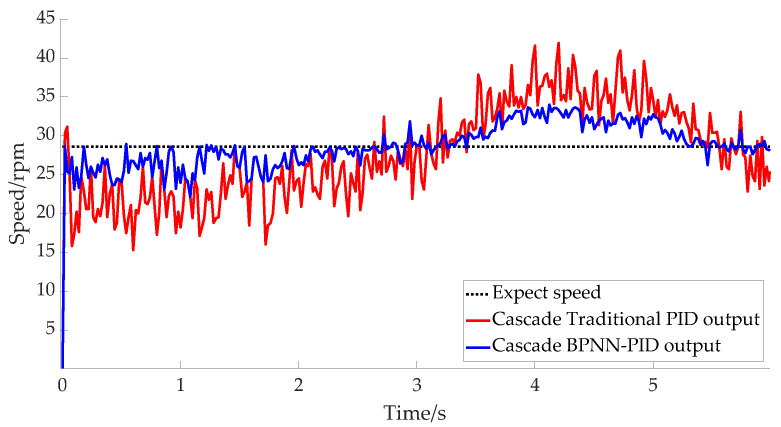
Responses of main motor rotating mechanisms.

The experimental test of the robot sampled the motor speed of each mechanism. The expected speed of the rotating mechanism of the main motor was set at 28.6 rpm. Figure 10 shows the response curve of the cascaded traditional PID algorithm and the cascaded BPNN–PID method. It can be seen from the figure that the speed response curve range of the cascade PID output was [15, 40] rpm, and the maximum error floating rate was 47.5%; in contrast, the speed response curve range of the cascade BPNN–PID output was [23, 33] rpm, and the maximum error floating rate was 19.5%. This further proves the superior performance and strong robustness of the BPNN adjustment algorithm.

**Figure 11:**
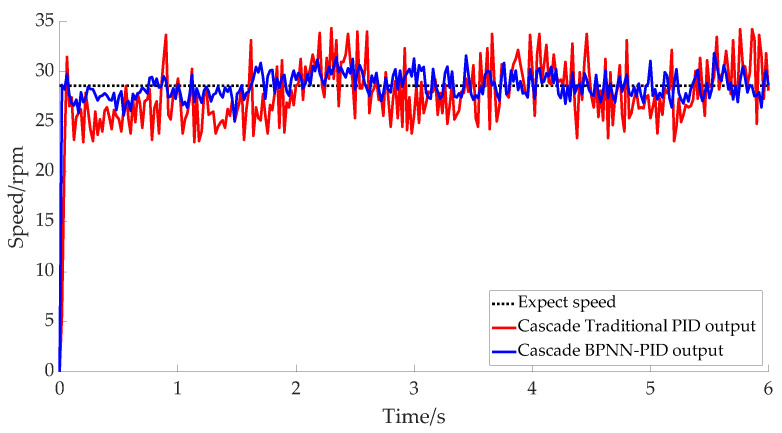
V-type collet-lifting mechanism actual speed outputs.

The V-type collet was expanded and the cable was clamped after adjusting the motor speed of the V-type collet lifting mechanism to 28.6 rpm. The speed response curves for the motor using the two controllers are shown in Figure 11, where it can be seen that the output response curve range of the BPNN–PID controller was [25, 30] rpm, and the maximum error floating rate was 12.5%, whereas the output range of the traditional PID response curve was [22, 34] rpm, and the maximum error floating rate was 23.1%. This also indicates that although the robot’s response using the BPNN–PID controller was better than that using the traditional PID controller, this advantage was not obvious. This is because in general, the actual speed output under the two controllers often fluctuated during the big gear rotation. When the tool rest and V-chuck lifting mechanism increased with the rotation of the gear, the speed was slightly slower. The gravitational effect made the velocity increase after the peak period until it finally reached the ideal level, while the external disturbance was relatively small.

**Figure 12:**
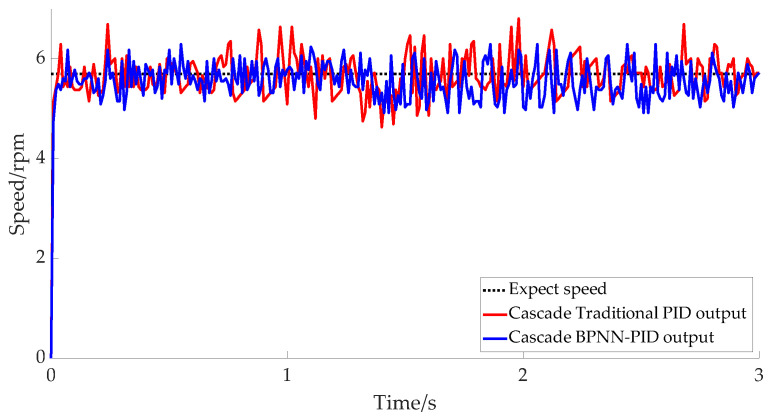
Tool rest lifting mechanism actual speed outputs.

The motor speed of the tool rest lifting mechanism was set at 5.7 rpm. The speed response over time in Figure 12 shows that the overall process of the tool rest lifting was relatively stable. The output speed range of the cascade BPNN–PID method was [5.1, 6.2] rpm, and the maximum error floating rate was 10.5%, whereas that of cascade PID method was [4.9, 6.9] rpm. The maximum error fluctuation rate was 21.1%, which further indicates that the cascade BPNN–PID method is superior to the cascade PID method in terms of fluctuation and velocity response. The cascade BPNN–PID controller was more efficient than the traditional cascade PID controller.

We performed 25 cable-stripping tests using the cascade BPNN–PID algorithm, measuring the length of peeled wire, the time it took to strip the cable, and the degree of cable damage. Table 2 displays the results of the wire-stripping test. The findings of 25 group tests reveal the stripping times of five group lengths (5 cm, 10 cm, 15 cm, 20 cm, and 25 cm) with an average stripping speed of 6.25 cm/min. The system’s stability could be preserved as a result of the moderate stripping pace. At the same time, analysis of the damage situation indicated that the wire stripper would also be slightly damaged. This was because the high voltage cable was excessively bent during installation, resulting in a delay in tool adjustment; this needs to be improved in the future. However, analysis of the task completion rate indicated that the integrity rate reached higher than 70%, further attesting to the robot’s high reliability; in addition, the cable damage effect showed that the damage was tolerable.

## 5. Conclusions

In this paper, a cable-stripping robot for distribution lines was designed; the robot is light in weight, small in size, and easy to carry, which is helpful for power workers who perform outdoor work. To improve the working stability of the cable-stripping robot, its cascade BPNN–PID control scheme was investigated, and experiments were carried out to assess its performance. The cable-stripping robot’s tests were used to assess the mechanical structure’s logic, the system’s hardware and software, and the adaptive control algorithm’s dependability. The cable-stripping robot helped increase live working efficiency and advanced smart grid development.

## Figures and Tables

**Table 1:** Key parameters of the cable-stripping robot.

Name	Parameter
Weight	3 kg
Dimensions	230 mm × 160 mm × 210 mm
Maximum continuous working hours	45 min
Maximum communication distance	500 m
Maximum cutting speed	5 cm/min

**Table 2:** Test data for wire-stripping experiments.

Serial Number	Stripping Length/cm	Stripping Time	Cable Damage
1	5	48″	Intact
2	5	50″	Intact
3	5	52″	Slight damage
4	5	55″	Slight damage
5	5	49″	Intact
6	10	1′48″	Intact
7	10	1′52″	Slight damage
8	10	1′50″	Intact
9	10	1′59″	Intact
10	10	1′46″	Intact
11	15	2′25″	Intact
12	15	2′23″	Intact
13	15	2′26″	Intact
14	15	2′21″	Slight damage
15	15	2′27″	Intact
16	20	3′15″	Intact
17	20	3′08″	Intact
18	20	3′10″	Intact
19	20	3′12″	Slight damage
20	20	3′08″	Intact
21	25	4′05″	Slight damage
22	25	4′02″	Slight damage
23	25	3′59″	Intact
24	25	4′01″	Intact
25	25	3′59″	Intact

## Data Availability

Data presented in this study are available on request from the corresponding author.
